# Sexual dimorphism in *Drosophila melanogaster* survival of *Beauveria bassiana* infection depends on core immune signaling

**DOI:** 10.1038/s41598-018-30527-1

**Published:** 2018-08-21

**Authors:** Parvin Shahrestani, Moria Chambers, John Vandenberg, Kelly Garcia, Glen Malaret, Pratik Chowdhury, Yonathan Estrella, Ming Zhu, Brian P. Lazzaro

**Affiliations:** 1 Department of Entomology, Cornell University, 129 Garden Avenue, Ithaca, NY USA; 2 USDA ARS Emerging Pests and Pathogens Research Unit, Robert W. Holley Center for Agriculture & Health, Tower Road, Ithaca, NY 14853 USA; 3 Present Address: Department of Biological Science, California State University Fullerton, 800 North State College Blvd., Fullerton, CA 92831-3599 USA; 4 Present Address: Department of Biology, Bucknell University, 1 Dent Drive, Lewisburg, PA USA

## Abstract

In many animal species, females and males differ in physiology, lifespan, and immune function. The magnitude and direction of the sexual dimorphism in immune function varies greatly and the genetic and mechanistic bases for this dimorphism are often unknown. Here we show that *Drosophila melanogaster* females are more likely than males to die from infection with several strains of the fungal entomopathogen *Beauveria bassiana*. The sexual dimorphism is not exclusively due to barrier defenses and persists when flies are inoculated by injection as well as by surface exposure. Loss of function mutations of Toll pathway genes remove the dimorphism in survivorship. Surprisingly, loss of function mutation of *relish*, a gene in the Imd pathway, also removes the dimorphism, but the dimorphism persists in flies carrying other Imd pathway mutations. The robust sexual dimorphism in *D. melanogaster* survival to *B. bassiana* presents opportunities to further dissect its mechanistic details, with applications for biological control of insect vectors of human disease and insect crop pests.

## Introduction

Physiology and lifespan differ between females and males across many animal species^1,2^. Dimorphism between the sexes can also affect host-parasite interactions, and sex differences in susceptibility to infection have been previously reported^3–9^. However, the mechanistic and genetic bases for sex differences in infection-susceptibility have not been well-characterized.

The laboratory fruit fly, *Drosophila melanogaster*, can be used as a model organism to study sex differences in immune defense*. D. melanogaster* is an established genetic model that shares innate immune response pathways with mammals^10–14^. Innate immunity is the first line of defense against pathogenic infections. Most immune-defense studies in flies and in mammals have focused on responses to bacterial infections, while defense against fungal infections remains understudied in comparison. Improving our understanding of immune defense against fungal infections, especially in insect hosts, has implications for the biological control of mosquito vectors of human disease and insect crop pests. The fungal entomopathogen used in our study, *Beauveria bassiana*, is used in biological control^15–19^. Understanding sexual dimorphism in insect susceptibility to *B. bassiana* can aid biological control efforts, and may be particularly useful for biological control of female mosquitoes that vector human disease.

In resisting fungal infections, there are two broad categories of defense that could be sexually dimorphic in insects. There is an initial barrier defense followed by the internal immune response after the fungus has penetrated the cuticle. Sex differences in susceptibility to infection may depend on barrier defenses such as cuticle integrity and on behavioral defenses such as grooming. Indeed, Taylor and Kimbrell^20^ observed lower survival in *D. melanogaster* females than males after *B. bassiana* spores were introduced to their cuticles. It is possible that this sex difference in survivorship arose through cuticular differences, given that male *D. melanogaster* have thicker cuticles that might have evolved because of their need for extra protection in fights with other males^21^ in which males are likely to be wounded. To resolve the relative roles of barrier defense and systemic immunity, our study employs two different inoculation techniques: inoculation by spray, which can be impacted by barrier defenses and grooming, and inoculation by injection, which transfers spores directly into the hemolymph.

When fungal spores come into contact with fly epithelia, they encounter diverse antimicrobial peptides that are constitutively expressed and may impact spore germination as well as the ultimate survival of the fly^22^. In addition, the trachea, reproductive tract, and gut can mount local immune responses^11^. Once the fungus enters the fly cavity, death by infection may result from the fungus consuming nutrients in the hemolymph^23^ or from toxins released by the fungus^24^. The fly may respond to penetration by the fungus through systemic immune responses, which include antimicrobial peptide expression, phenoloxidase activity, microbial phagocytosis by hemocytes, and hemocyte abundance. Our investigation focuses on the role of Toll and Imd pathways, which are key regulators of the immune response and are largely responsible for the systemic inducible expression of antimicrobial peptides^10–12^. Taylor and Kimbrell^20^ observed that mutating genes in the Toll and Imd pathways did not eliminate sexual dimorphism in defense against fungal infection. However, as our preliminary results opposed those prior findings, we tested the relative importance of these pathways by assessing whether mutants in different components of the signaling pathways demonstrated sexual dimorphism in their survival to fungal infection.

In our study, we found that *D. melanogaster* females are more susceptible than males to several strains of *B. bassiana*. The sexually dimorphic defense is not exclusively due to barrier defenses and persists even when flies are inoculated by injection. Loss of function mutations of Toll pathway genes remove the dimorphism in susceptibility. Interestingly, loss of function mutation of Relish, a gene in the Imd pathway, also removes the dimorphism, but the dimorphism is retained in other Imd pathway mutants.

## Methods

### Drosophila melanogaster hosts

All *D. melanogaster* lines and populations used in our study were maintained on glucose-yeast medium (100 g/L yeast, 100 g/L glucose, 1% Drosophila agar), at ~25 °C and 12light:12dark cycles. The wild-type fly lines were Canton-S (CS, Bloomington stock # 1), Oregon R (OrR, Bloomington stock # 5), and *w*^1118^ (Bloomington stock # 6326). The Imd pathway mutants were *imd*^10191^ ^25^, *dTAK1*^*D10*^ ^26^, *relish*^*E20*^ ^27^, and *PGRP-LE*. The Toll pathway mutants used were *spz*^*rm7*^ ^28^, *modsp*^29^ and *persephone*^30^.

The outbred population, PopC3, was derived from the Global Diversity Lines^31,32^ obtained from the laboratory of Andy Clark at Cornell University. Ninety-two *D. melanogaster* lines from five populations (China (Beijing), USA (Ithaca), Netherlands (Houten), Tasmania, and Zimbabwe (Sengwa and Harare)) were established from isofemale lines and then inbred for 12 generations. These lines were then crossed within-population in round-robin and reciprocal round-robin crosses. In the subsequent generation, the F1 from these crosses were randomly paired in between-source population crosses to create the F2 generation. Then two males and two non-virgin females from each of the F2 crosses were placed in each of ten fly bottles to establish a megapopulation that was ancestral to PopC3. The megapopulation was subsequently maintained with non-overlapping ~14-day generations at population size of ~2000 flies in population cages at 25 °C with 12:12 light/dark cycle for ~1 year. This population was then grown to a larger population size of 16,000 flies over several generations. PopC3 was then separated from the megapopulation as a random sample of 2000 flies and subsequently maintained on ~28 day generation cycles in population cages.

To generate RNAi knockdown lines, virgin females from the c564-gal4 driver line (Bloomington Stock # 6982) were collected. These were maintained for 3 days to ensure their virgin status. Then ten virgin c564-Gal4 flies were crossed to ten male flies of either *w*^1118^ (Bloomington stock #6326) or UAS-rel RNAi (Vienna Stock # 108469) in vials. Adults were flipped to new vials every two days to ensure that offspring would be age-matched for the injection assays.

**Figure 1:**
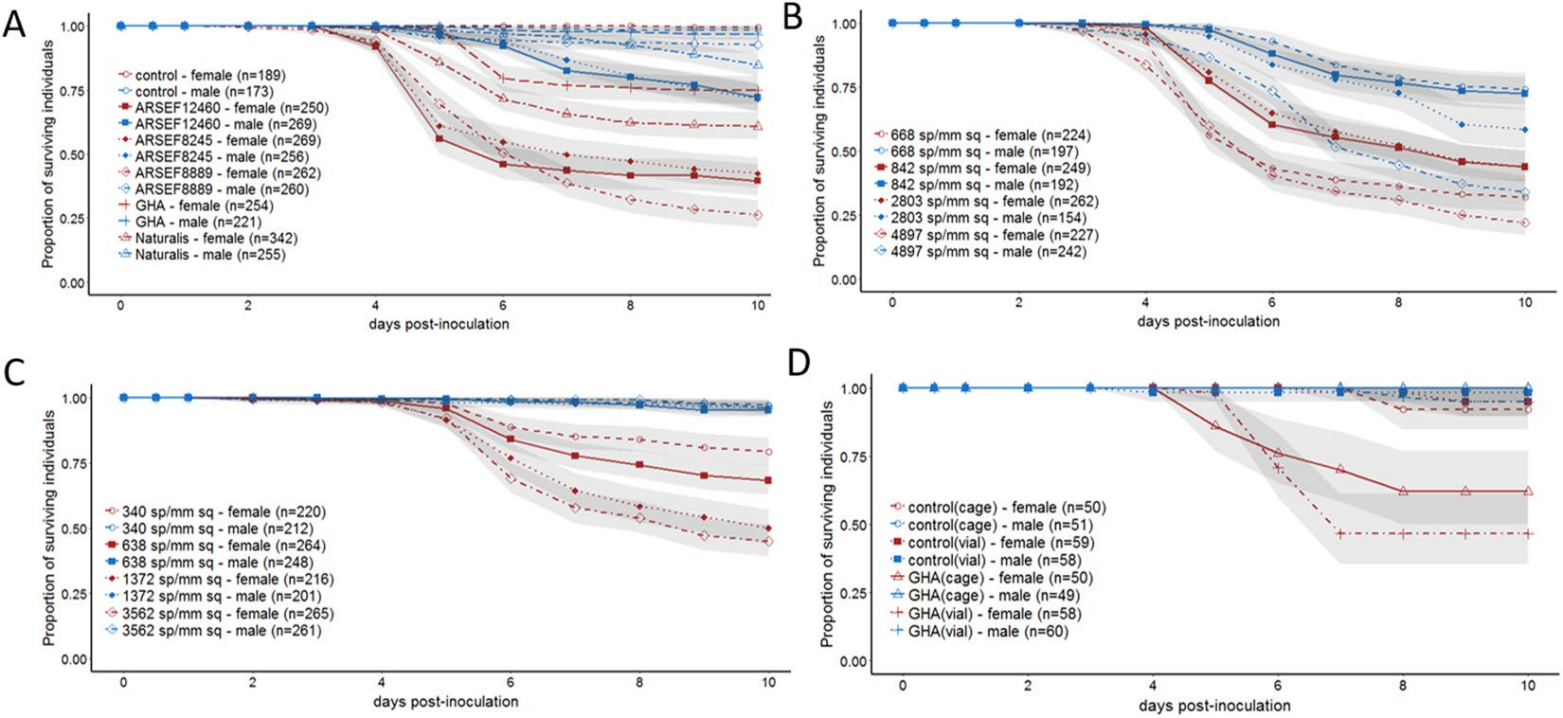
Survival after infection with diverse B. bassiana strains and conditions. Canton S (**A**–**C**) and PopC3 (**D**) flies were infected with *B. bassiana* using the spray method. Survival after inoculation with (**A**) a range of *B. bassiana* strains – control (no infection), ARSEF 12460, ARSEF 8245, ARSEF 8889, and GHA, (**B**) ARSEF 12460 at multiple doses, (**C**) GHA at multiple doses, (**D**) GHA and housed in either vial or cages post infection. Statistics assessing the impact of sex on survival for each condition are reported in Table S1.

**Figure 2:**
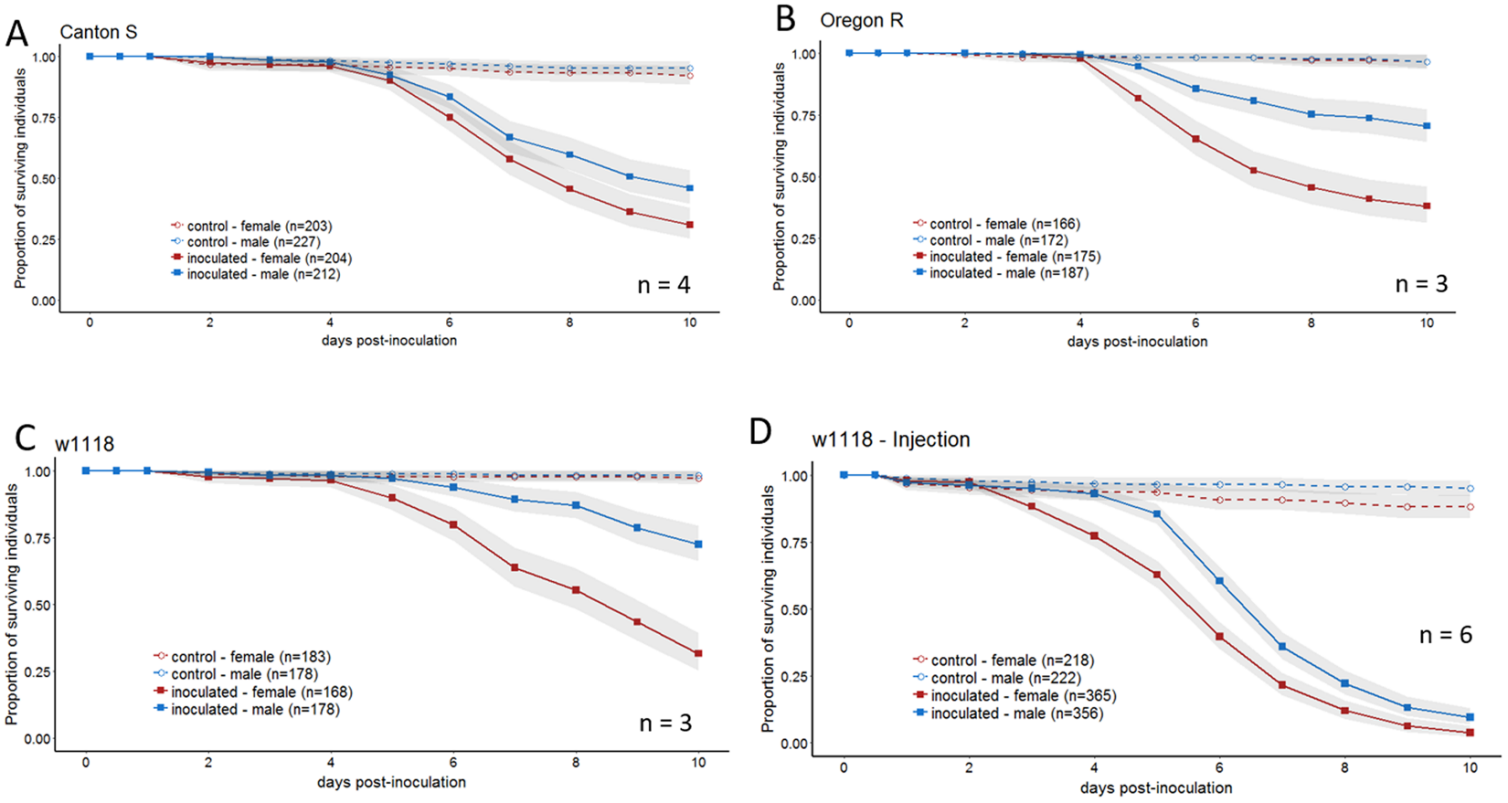
Dimorphism persists in common laboratory strains and when the cuticle is bypassed via injection. Flies were sprayed (**A**–**C**) or injected (**D**) with GHA or silwet, and monitored for survival for 10 days. Common laboratory fly lines (**A**) Canton S, (**B**) Oregon R, (**C**,**D**) w1118 were tracked for survival post-inoculation. Graphs are a combination of multiple independent replicates (n) indicated on each panel. Statistics assessing the impact of sex on survival for each condition are reported in Table S2.

**Figure 3:**
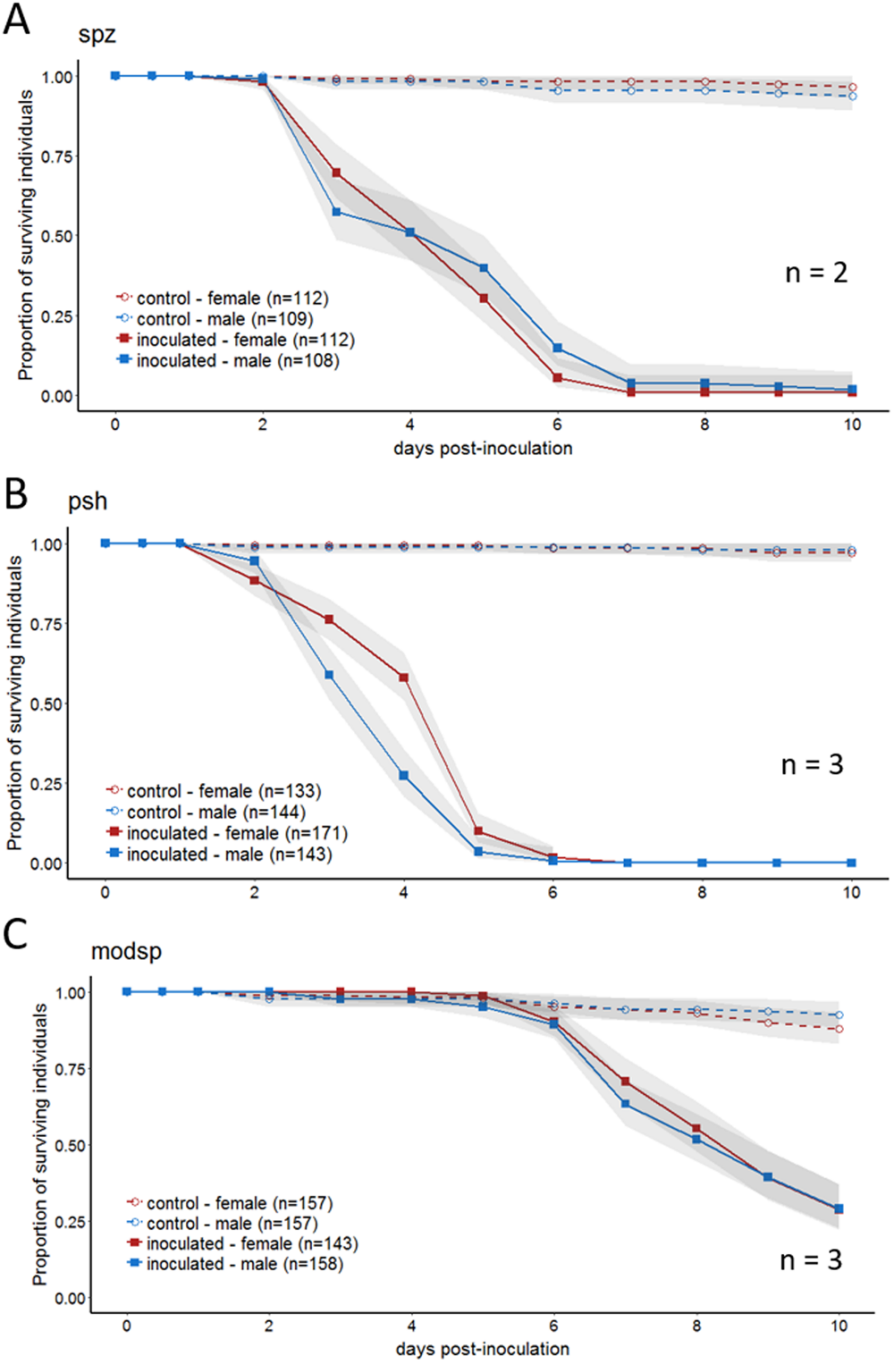
Mutations in the toll pathway alter the sexual dimorphism. Survival after spraying flies with GHA was monitored for 10 days in (**A**) *spz* mutants (**B**) *psh* mutants (**C**) and *modsp* mutants. Graphs are a combination of multiple independent replicates (n) indicated on each panel. Statistics assessing the impact of sex on survival for each line is reported in Table S3.

**Figure 4:**
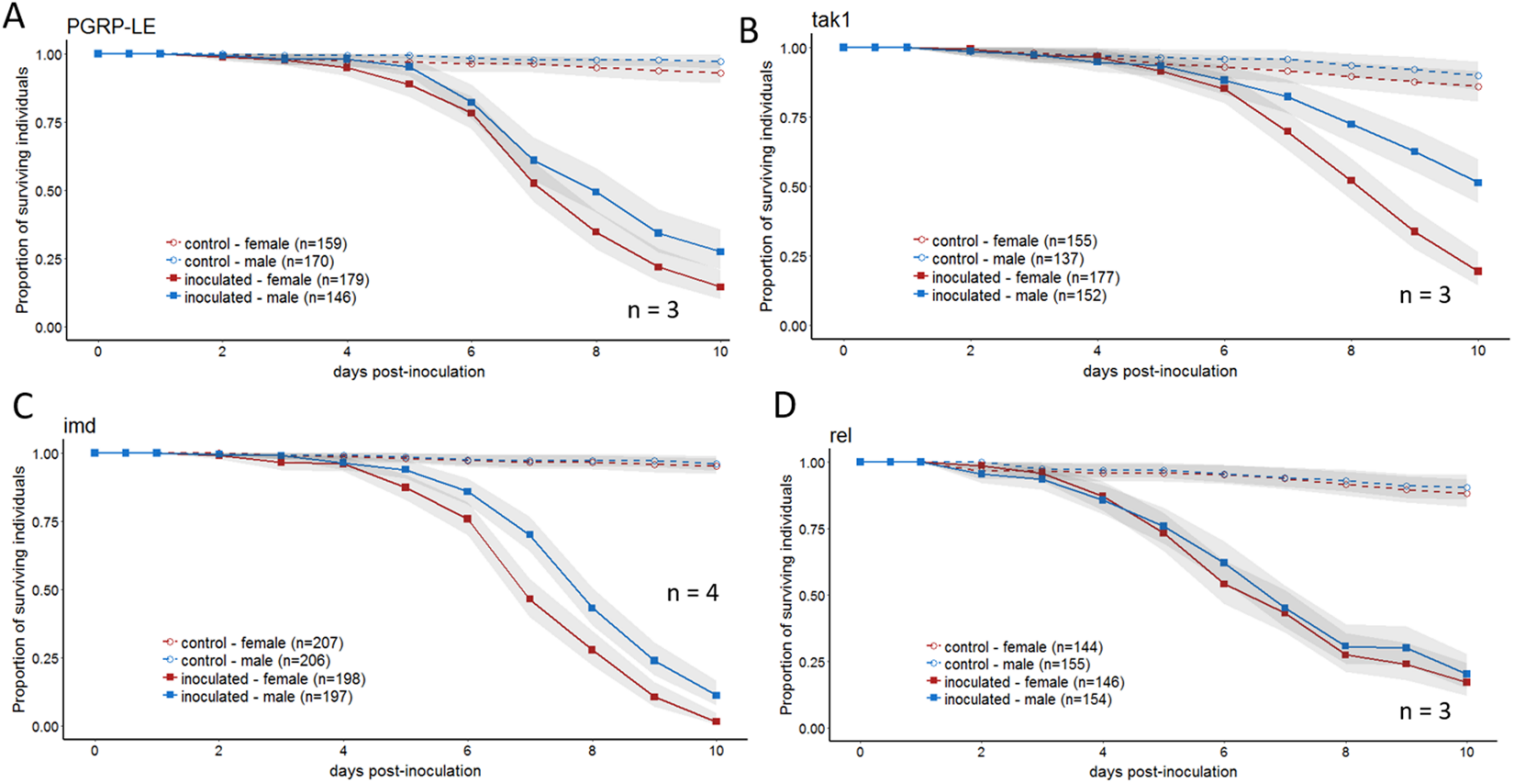
Impact of Imd pathway on sexual dimorphism. Survival after spraying flies with GHA was monitored for 10 days in (**A**) *PGRP-LE* mutants, (**B**) *tak1* mutants, (**C**) *imd* mutants, (**D**) and *relish* mutants. Graphs are a combination of multiple independent replicates (n) indicated on each panel. Statistics assessing the impact of sex on survival for each line is reported in Table S4.

**Figure 5:**
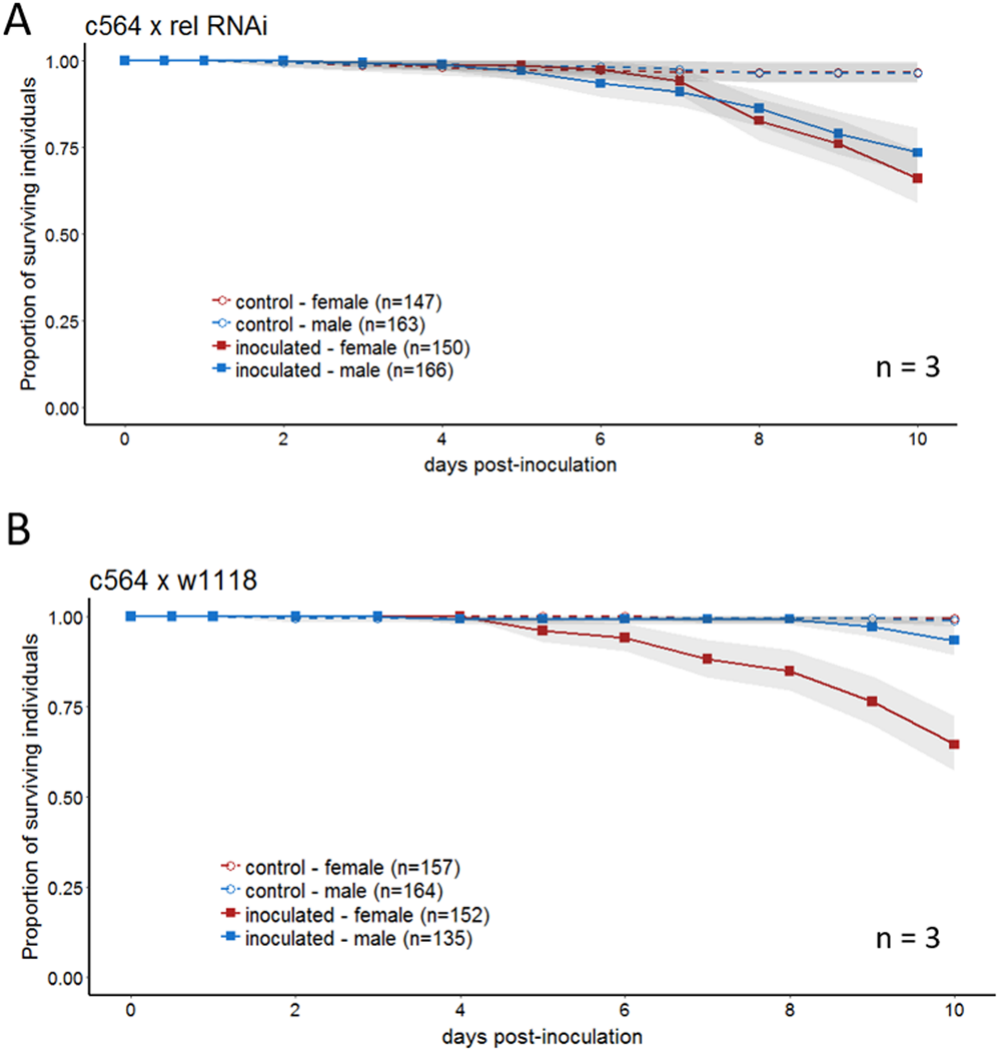
RNAi confirms the impact of relish on sexual dimorphism. Fatbody driver flies (c564) were crossed to either (**A**) a fly line containing a relish RNAi construct or (**B**) the control background. Offspring of these crosses were sprayed with GHA and monitored for survival for 10 days. Graphs are a combination of multiple independent replicates (n) indicated on each panel. Statistics assessing the impact of sex on survival for each line is reported in Table S4.

The experiments shown in Fig. 1A–C were replicated, but flies were reared at the same time and sprayed with the same fungal suspension, so the replication is across the cages in which the flies were maintained. The experiment in Fig. 1D was not replicated. The experiments presented in Figs 2–5 were replicated with independent rearing of flies and independently prepared fungal suspensions. The number of replicates for each experiment are shown on the figures and in the supplementary tables.

### Fungal pathogens

We used five strains of the entomopathogenic fungus *Beauveria bassiana* obtained from ARSEF (USDA Agricultural Research Service Collection of Entomopathogenic Fungi, Ithaca, NY). ARSEF 12460 was isolated from one *D. melanogaster* that was infected with ARSEF 8246 (originally isolated from the shorefly, *Scatella tenuicosta*). GHA is based on ARSEF 6444, isolated from the migratory locust, *Locusta migratoria*, and is used as a technical spore powder produced by Mycotech, Inc. (now Bioworks, Inc., Victor NY, lot number TGA1-96-06B). Naturalis (Troy Biosciences, Inc.) is based on ARSEF 7795, originally isolated from the Boll weevil, *Anthonomis grandis*. ARSEF 8245 was isolated from *S. tenuicosta* and ARSEF 8889 was isolated from the lygus bug, *Lygus linearis*. The fungal spores were cultivated on SDAY medium and spores were scraped from the plate surface after two weeks incubation in darkness at 25 °C.

### Fungal inoculation by spray method

To prepare a fungal suspension, 0.34 g of *Beauveria bassiana* spores were suspended in 25 ml of 0.03% Silwet L-77 (Loveland Industries, Inc., Greeley, CO). The viabilities of the fungi were verified as >90% by germinating samples of spores on SDAY. The approximate dose of *B. bassiana* was estimated by placing a microscope slide adjacent to the flies, then suspending this slide in 0.03% silwet and using a hemocytometer to count the spores^33^. Dosages ranged from 340 spores/mm^2^ to 4897 spores/mm^2^ (Fig. 1B,C). In experiments where dosage was not a variable, the dose used was ~3500 spores/mm^2^.

In preparation for the assays, at day two from eclosion, flies were moved to 8-dram vials containing ~8 mL of medium, at densities of 5 females and 5 males per vial. Then, at 5–8 days post eclosion, flies were either sprayed with the fungal inoculate or with a silwet control. We used young flies to avoid the potentially confounding effects of immune senescence. For each spray, approximately 50 female and 50 male flies were anesthetized using CO_2_, placed on ice for the duration of inoculation (<3 min), and inoculated using a spray tower^34^. Inoculated flies were then moved to ~2050 ml mesh population cages, fed with a small Petri plate of fly medium, and kept at ~100% humidity for 24 hours. In high humidity conditions, fungal conidia germinate and the hypha penetrate the insect cuticle and grow in the hemocoel^35^.

Flies that died in the first 24 hours post inoculation were discarded, but this handling loss did not exceed 4%. For the following 9 days, the humidity was lowered to 60–70% and the food dish was replaced daily. Dead flies were removed each day to avoid secondary inoculation of live flies by spores on the cuticles of the deceased flies and to record the numbers of dead females and males. At the end of the tenth day, remaining live flies were sexed and counted.

### Fungal inoculation by injection

Fungal suspension was prepared as described above (0.34 g of *Beauveria bassiana* spores in 25 ml of 0.03% silwet). This suspension was diluted 1:1000 in 0.03% silwet and then 23 nL was injected into the abdomen of each fly using a Nanoject II (Drummond, www.drummondsci.com). This corresponds to approximately 30–70 spores per fly. Control flies were injected with 23 nL of 0.03% silwet. Flies were anesthetized with light CO_2_ for less than five minutes during the injection procedure. All controls were exposed to CO_2_ for the same amount of time and no CO_2_-induced mortality was observed. Infected flies were kept at 100% humidity for 24 hours to allow the fungus to germinate and then maintained at 25 °C in an incubator with a 12:12 hour light-dark cycle. Mortality was monitored daily and flies were transferred to fresh vials every two days.

### Analysis of variance

Statistical analyses of survival were performed using R^36^; [http://www.r-project.org/]. Survival curves are plotted as Kaplan-Meier plots and a model reflecting the contribution of experimental factors was built using the Cox Proportional Hazards (*coxph*) process within the package “Survival” in R. Each factor is incorporated in sequence and factors are listed in order of inclusion in result tables. Sources of variation were assessed by subsequent ANOVA.

The factors in each model below are defined for the model in which they first appear. Interactions between factors are represented as Factor 1 × Factor 2. All factors were considered to contribute fixed effects. All models tested the role of sex (S: male or female) on survival and additional factors were included as needed.

Model A tested the effect of fungal strains (F) on survival post-infection.

$${\rm{Model}}\,{\rm{A}}:\,{\rm{coxph}}\,({\rm{status}},{\rm{time}})={\rm{F}}+{\rm{S}}+({\rm{F}}\times {\rm{S}})$$

Model B determined the impact of infectious dose (ID) on survival after infection. The infectious dose variable was input as a quantitative variable in the model as the number of spore/mm^2^.

$${\rm{Model}}\,{\rm{B}}:\,{\rm{coxph}}\,({\rm{survival}},{\rm{time}})={\rm{ID}}+{\rm{S}}+({\rm{ID}}\times {\rm{S}})$$

Model C assessed the impact of housing *D. melanogaster* in smaller vials versus larger cages after infection. Flies were housed in different containers (C: cages or vials).

$${\rm{Model}}\,{\rm{C}}:\,{\rm{coxph}}\,({\rm{survival}},{\rm{time}})={\rm{C}}+{\rm{S}}+({\rm{C}}\times {\rm{S}})$$

Model D assessed the impact of sex on infection in various fly lines. A factor for replicate (R) was included to control for variation between independent experiments and the interaction term R x S was used to assess the robustness of the sexual dimorphism. When there was a significant interaction term, the results were examined on a per replicate basis to evaluate whether the direction and presence of the phenotype were dependent on replicate (data not shown).

$${\rm{Model}}\,{\rm{D}}:\,{\rm{coxph}}\,({\rm{survival}},{\rm{time}})={\rm{R}}+{\rm{S}}+({\rm{R}}\times {\rm{S}})$$

## Results

### Sexual dimorphism in survival to *B. bassiana* is robust and persists across different fungal strains, doses, delivery methods, and housing conditions

To determine whether *D. melanogaster* are consistently sexually dimorphic in their response to inoculation with *B. bassiana*, we tested several commonly used bio-control strains for their ability to kill male and female Canton-S *D. melanogaster*. All strains that were tested killed significantly more females than males (Sex: p < 0.001), although the extent of the dimorphism depended on the strain (Fungal Strain * Sex: p < 0.001, Fig. 1A, results from complete linear model found in Table S1). To determine whether the dimorphism was dose-dependent in two commonly used *B. bassiana* strains, ARSEF12460 and GHA were sprayed onto Canton-S *D. melanogaster* with suspensions of various dilutions. Both strains of *B. bassiana* cause a sexual dimorphism in survival across all doses, with more females dying than males for each condition (Sex: p < 0.0001, Fig. 1B,C, results from complete linear model found in Table S1). However, the strength of the dimorphism was dependent on dose, with higher doses showing a more robust difference due to a strong dose response in male survival while female survival remained relatively constant across doses (Sex*Dose, p = 0.03, results from complete linear model found in Table S1). To establish whether the post-inoculation housing conditions impacted the sexual dimorphism in *D. melanogaster* survivorship and to determine whether the dimorphism extended to a genetically diverse population, we inoculated the *D. melanogaster* population PopC3 with GHA and tracked post-infection survival in both vials and population cages. The sexual dimorphism was highly significant in this genetically diverse population (Sex: p < 0.0001, Fig. 1D, results from complete linear model found in Table S1) and the difference in container types contributed no significant effect on survival (Container*Sex: p = 0.12, results from complete linear model found in Table S1). We tested whether the sexual dimorphism in survival extended to some commonly used laboratory strains of *D. melanogaster*, and found that Canton-S, Oregon-R and *w*^1118^ all exhibited sexual dimorphism in survival, with females dying more than males (Sex: p < 0.0001, Fig. 2, results from complete linear model found in Table S2).

### Sexual dimorphism to *B. bassiana* does not depend entirely on cuticular defense

Initial observations of the flies infected by the spray method revealed increased grooming by both male and female flies. However, flies often failed to groom hard-to-reach areas such as the lower dorsal thorax [also seen in ^20^] and the joints of the limbs. We did not notice an obvious sexual dimorphism in grooming behavior. Female flies are larger than male flies in comparisons of mass^37^. Given that *D. melanogaster* females are larger than males, in the spray method each female on average received more spores on her cuticle than each male. However, this size difference is unlikely to be a cause of the increased susceptibility to infection in females because females inoculated at the lowest dose in our dose-response assays are still more susceptible than males inoculated at the highest dose despite receiving about 10-fold fewer spores (Fig. 1B,C).

To test whether the sexual dimorphism in survivorship was entirely due to differences in cuticular defenses or grooming behaviors, we measured survivorship after injection of spores directly into the hemolymph. We found that the sexual dimorphism persisted even when *B. bassiana* was injected into the hemolymph of *D. melanogaster* (Sex: p < 0.001, Fig. 2D, results from complete linear model found in Table S2), suggesting that the sexual dimorphism is not exclusively due to barrier defenses and probably also involves the internal immune response. The results of our injection assay also provide further rejection of the notion that size differences between males and females could cause the observed difference in immune defense. In the injection assays, the same volume of suspension was introduced to both sexes, so each female received fewer spores per body mass than each male, yet females were still more susceptible to infection (Fig. 2D).

### Mutations in Toll pathway ablate or reverse the sexual dimorphism

The Toll pathway responds to fungal molecules and products, so we inoculated three different Toll pathway mutants to determine whether the Toll pathway was essential for the sexual dimorphism in survival. Mutants in *spz*^*rm7*^, which encodes the ligand that directly binds Toll, as well as two upstream activators, *persephone* and*,modSP*, all significantly impacted the sexual dimorphism (Fig. 3, Table S3). Mutants in *spz*^*rm7*^ and *modSP* no longer exhibited any significant difference in survival based on sex (Sex: p > 0.05, Fig. 3A,C); while mutants in *persephone* demonstrated a sexual dimorphism but in the opposite direction, with males dying more quickly than females after infection (Sex: p = 0.0007, Fig. 3B, results from complete linear model found in Table S3).

### Upstream mutations in the Imd pathway do not impact sexual dimorphism, but knockout and knockdown of Relish ablates the sexual dimorphism

The Imd pathway is a second pathway that regulates the *D. melanogaster* humoral immune response, although it is not thought to be generally required for resistance to fungal infection^11^. To determine whether the Imd pathway was essential for the sexual dimorphism in survival, we inoculated flies carrying mutations in four different genes in the pathway (*PGRP-LE*, *imd*, *tak1*, and *Relish)*. Flies with mutated *PGRP-LE*, *imd* and *tak1* all demonstrated a significant sexual dimorphism with females dying more quickly than males (Sex for all mutants: p < 0.005, Fig. 4, Table S4). *D. melanogaster* mutant for *Relish*, the terminal transcription factor in the Imd pathway, exhibited no sexual dimorphism in survival to *B. bassiana* infection (Sex: p = 0.48, Fig. 4D, results from complete linear model found in Table S4). This ablation of the sexual dimorphism was confirmed using RNAi knockdown of *Relish* in the immune tissues of the fly (fatbody, hemocytes and oenocytes), resulting in flies that no longer demonstrated the sexual dimorphism (Fig. 5, Sex: p = 0.12). Control flies that were heterozygous for the transgenic driver but lacked any RNAi construct maintained the dimorphism (Sex: p < 0.0001, Fig. 5, results from complete model found in Table S4).

## Discussion

We observed a robust sexual dimorphism in *D. melanogaster* survival of *B. bassiana* infection. The dimorphism was observed whether flies were inoculated by spraying of a fungal suspension on the cuticle or by injection of fungal spores into the hemocoel. Females were more susceptible to infection than males under both inoculation methods (Fig. 2). While the direction of the dimorphism was consistent across fungal strains, doses, inoculation method, and wild-type fly lines, the magnitude of the dimorphism varied across experiments. This is possibly due to sensitivity of *B. bassiana* infection to minor changes in environment, especially humidity. We maintained all laboratory conditions as consistently as possible. However, some seasonal variation was inevitable, potentially leading to assays performed at different times of the year having some variation in the magnitude of the dimorphism.

The sexual dimorphism was eliminated when critical genes in the immune response were mutated. This leads us to believe that even if barrier immunity (cuticular thickness, differences in grooming, etc.) makes contribution to the dimorphism, the internal immune response is the primary determinant of the sexual dimorphism. The Toll pathway helps fight fungi and Gram positive bacteria [reviewed in^38^]. We found that the Toll pathway played an essential role in the sexual dimorphism. The Toll pathway is involved in defense against fungal pathogens, and as expected, flies that were mutant for Toll pathway components died more quickly than wild type controls during our experiments, regardless of sex (Figs 2 and 3). With loss-of-function mutations of Toll pathway genes *spz*^*rm7*^ and *modSP*, the sexual dimorphism disappeared and males no longer had a survival advantage compared to females. These results contrast to those of Taylor and Kimbrell^20^, who found that females were more susceptible to *B. bassiana* infection, even in *spz* mutants. The inoculation method used by those authors was different than ours in that, in their study, anesthetized flies were lightly shaken in a Petri plate containing fungal spores. We have no reason, however, to believe that this delivery method should produce a dramatically different outcome with respect to sexual dimorphism. Additionally, infected flies in Taylor and Kimbrell^20^ were maintained in vials instead of in cages. However, in our experiment comparing housing in vials versus cages, there was no significant change in the dimorphism phenotype, which leads us to think that housing post-inoculation was not likely to cause the inconsistency between our results with *spz*^*rm7*^ mutants and those of Taylor and Kimbrell^20^. While it remains possible that differences in rearing media or other environmental factors could have contributed to our differing results, we are unable to pinpoint the reason for the very different results of our study compared to Taylor and Kimbrell^20^. We observed a reversal in the sexual dimorphism in *persephone* mutants, with males becoming more susceptible than females (Fig. 3B). This may indicate that there might be other underlying sexual dimorphisms in immune defense that can be revealed by removal or weakening of primary immune mechanisms.

The Imd pathway is traditionally thought to help fight Gram-negative infections^10–12,39^. Therefore, the Imd pathway is not expected to influence the sexual dimorphism in susceptibility to fungal infection. Indeed, Taylor and Kimbrell^20^ showed that mutations of two *imd* alleles (*imd1* and *P*-*imd*) did not affect sexual dimorphism in susceptibility to *B. bassiana*. However, these mutants did have reduced survival after inoculation compared to wildtype flies^20^. Others have also shown that ablating Imd signaling has detrimental effects on overall fungal immunity^40–42^.

We tested four Imd pathway mutants and saw that the dimorphism persisted in three of these: *PGRP-LE, tak1* and *imd* (Fig. 4A–C). However, surprisingly, the dimorphism disappeared entirely in *Relish* mutants (Fig. 4D). The Drosophila NF-κB protein, Relish, is a transcription factor in the Imd pathway, leading to AMP gene induction. The activation of Relish is thought to be controlled by the IKK complex, through mechanisms that either involve kinase activity or do not involve kinase activity^39^. But while Relish may be activated by more than one mechanism, there is no previous evidence to suggest that Relish could be activated independently of the Imd pathway. This is despite observations that infection by fungi can activate *Relish*^41^, and that Toll and Imd pathways work synergistically to activate an immune response in flies^42^. At this time, we have no obvious explanation for why sexual dimorphism in susceptibility to fungal infection disappears in *Relish* mutants but is not affected in the other Imd pathway mutants. However, given that the *Relish* mutant results were also replicated in RNAi knockdowns of *Relish* (Fig. 5), it appears possible that *Relish* may have an effect on the sexually dimorphic response to infection, independently of the rest of the Imd pathway.

Previous studies have suggested that both the Toll and Imd pathways may be involved in defense against fungal infection, in that double mutants of the two pathways have increased susceptibility to infection^42^ and some antimicrobial peptide inducing genes seem to be regulated by both pathways^27,40,41,43,44^. Moreover, some antimicrobial peptides have both antibacterial and antifungal activity^44–48^. In a previous experiment with the fungus *G. candidum*, Hedengren *et al*.^27^ suggested that the recognition of fungi happens upstream of *Relish*, and that Relish may regulate antifungal peptides such as Metchnikowin. It may be the case that *Relish* may acts as a key integrator of these signaling pathways.

When diverse animals are studied for sexual dimorphism in immune defense, there is no consistent “sicker sex” as has sometimes been suggested^49^. In some cases, females are better at fighting off infections, as seen in some mammals, fish, birds, reptiles, insects, copepods, and dioecious plants^9,20,50,51^. Other times, males survive infection better, as observed in several arthropods^8,52^ [and in the present study]. In humans, females are more susceptible to toxoplasmosis, amoebiasis, and giardiasis, while males are more susceptible to malaria and schostosomiasis^53–55^. These results suggest that sex differences in immune defense may depend on the specific pathogen and host combination, and may occur in response to variation in the availability of fitness-limiting resources. In other words, there may be sex-specific plasticity affecting the magnitude and direction of sexual dimorphism in immune defense^56^. We have described a consistent sexual dimorphism in *D. melanogaster* infected with *B. bassiana*, which can be used to explore the many factors affecting the direction and magnitude of this dimorphism.

## Electronic supplementary material

Supplementary file
