# Supplementary material for: Sexual dimorphism in Drosophila melanogaster survival of Beauveria bassiana infection depends on core immune signaling
Source: Sci Rep. 2018 Aug 21;8:12501. doi: 10.1038/s41598-018-30527-1 (PMC6104035; doi:10.1038/s41598-018-30527-1)
Supplement: Supplementary file 1 — Supplementary file [file 41598_2018_30527_MOESM1_ESM.docx]

**Supplementary Material**

**Sexual dimorphism in *Drosophila melanogaster* survival of *Beauveria bassiana* infection depends on core immune signaling**

Parvin Shahrestani^Ϯ#α*^, Moria Chambers ^Ϯϯα^, John Vandenberg^€^, Kelly Garcia ^Ϯ^, Glen Malaret ^Ϯ^, Pratik Chowdhury ^Ϯ^, Yonathan Estrella ^Ϯ^, Ming Zhu ^Ϯ^, Brian P. Lazzaro ^Ϯ^

^Ϯ^ Department of Entomology, Cornell University, 129 Garden Avenue, Ithaca, NY, USA

^#^ Current address: Department of Biological Science, California State University Fullerton, 800 North State College Blvd., Fullerton, CA 92831-3599

^ϯ^ Current address: Department of Biology, Bucknell University, 1 Dent Drive, Lewisburg, PA, USA

^€^ USDA ARS Emerging Pests and Pathogens Research Unit, Robert W. Holley Center for Agriculture & Health, Tower Road, Ithaca NY 14853

^α^ Equal contribution

^*^ To whom correspondence should be addressed.

**Supplemental Tables**

**Supplemental Table 1: Impact of factors on survival (Figure 1).**

|  |  |  |  |  |  |
| --- | --- | --- | --- | --- | --- |
| Panel | *B. bassiana* Strain | Factor | χ2 | Df | p-value |
| 1A | Various | fungal strain | 152.6 | 4 | **<0.0001** |
|  |  | sex | 450.8 | 1 | **<0.0001** |
|  |  | fungal strain x sex | 59.8 | 4 | **<0.0001** |
|  |  |  |  |  |  |
| 1B | ARSEF12460 | dose | 70.1 | 1 | **<0.0001** |
|  |  | sex | 129.8 | 1 | **<0.0001** |
|  |  | dose x sex | 25.2 | 1 | **<0.0001** |
|  |  |  |  |  |  |
| 1C | GHA | dose | 36.4 | 1 | **<0.0001** |
|  |  | sex | 417.8 | 1 | **<0.0001** |
|  |  | dose x sex | 4.6 | 1 | 0.03 |
|  |  |  |  |  |  |
| 1D | GHA | container | 2.4 | 1 | 0.12 |
|  |  | sex | 67.0 | 1 | **<0.0001** |
|  |  | container x sex | 2.5 | 1 | 0.12 |

Flies were inoculated with *Beauveria bassiana* (various strains and doses) and the survival of each sex was recorded for ten days (Fig. 1). We used a Cox Proportional Hazards model (Model A, B & C, see Materials and Methods) followed by ANOVA to assess the contribution of factors to overall mortality.

**Supplemental Table 2: Effect of sex on mortality in common laboratory fly lines (Figure 2).**

|  |  |  |  |  |  |
| --- | --- | --- | --- | --- | --- |
| Fly Line | Infection method | Factor | χ2 | Df | p-value |
| Canton S | spray | replicate | 24.1 | 3 | **<0.0001** |
|  |  | sex | 11.0 | 1 | **<0.0001** |
|  |  | replicate x sex | 8.7 | 3 | 0.038 |
|  |  |  |  |  |  |
| Oregon R | spray | replicate | 1.9 | 2 | 0.40 |
|  |  | sex | 41.4 | 1 | **<0.0001** |
|  |  | replicate x sex | 4.3 | 2 | 0.12 |
|  |  |  |  |  |  |
| w1118 | spray | replicate | 4.9 | 2 | 0.09 |
|  |  | sex | 63.8 | 1 | **<0.0001** |
|  |  | replicate x sex | 9.9 | 2 | **0.007** |
|  |  |  |  |  |  |
|  | injection | replicate | 24.7 | 5 | **0.0001** |
|  |  | sex | 34.4 | 1 | **<0.0001** |
|  |  | replicate x sex | 8.2 | 5 | 0.15 |

Common laboratory lines of *Drosophila* were inoculated with *B. bassiana* (GHA strain) by either spray or injection and then monitored for survival for ten days post-inoculation (Figure 2). To assess the contribution of replicate and sex to survival, we used a Cox Proportional Hazards model (Model D, see Materials and Methods) followed by an ANOVA. Where sex x replicate is significant, the strength of the phenotype varies among the replicates, as opposed to the dimorphism disappearing in some replicates (data not shown).

**Supplemental Table 3: Effect of sex on mortality in fly lines mutant for Toll Pathway (Figure 3).**

|  |  |  |  |  |  |
| --- | --- | --- | --- | --- | --- |
| Fly Line | Infection method | Factor | χ2 | Df | p-value |
| spz | spray | replicate | 91.1 | 1 | **<0.0001** |
|  |  | sex | 2.0 | 1 | 0.16 |
|  |  | replicate x sex | 0.1 | 1 | 0.82 |
|  |  |  |  |  |  |
| psh | spray | replicate | 43.3 | 2 | **<0.0001** |
|  |  | sex | 11.3 | 1 | **0.0007** |
|  |  | replicate x sex | 2.0 | 2 | 0.35 |
|  |  |  |  |  |  |
| modsp | spray | replicate | 40.4 | 2 | **<0.0001** |
|  |  | sex | 0.05 | 1 | 0.47 |
|  |  | replicate x sex | 0.52 | 2 | 0.77 |

*Drosophila* lines that contain mutated versions of Toll pathway genes were inoculated with *B. bassiana* (GHA strain) by spray and then monitored for survival for ten days post-inoculation (Figure 3). To assess the contribution of replicate and sex to survival, we used a Cox Proportional Hazards model (Model D, see Materials and Methods) followed by an ANOVA.

**Supplemental Table 4: Effect of sex on mortality in fly lines mutant for Imd Pathway (Figure 4 & 5).**

|  |  |  |  |  |  |
| --- | --- | --- | --- | --- | --- |
| Fly Line | Infection method | Factor | χ2 | Df | p-value |
| PGRP-LE | spray | replicate | 25.9 | 2 | **<0.0001** |
|  |  | sex | 9.9 | 1 | **0.002** |
|  |  | replicate x sex | 7.9 | 2 | 0.02 |
|  |  |  |  |  |  |
| tak1 | spray | replicate | 25.4 | 2 | **<0.0001** |
|  |  | sex | 31.3 | 1 | **<0.0001** |
|  |  | replicate x sex | 20.3 | 2 | **<0.0001** |
|  |  |  |  |  |  |
| imd | spray | replicate | 15.4 | 3 | **0.001** |
|  |  | sex | 29.2 | 1 | **<0.0001** |
|  |  | replicate x sex | 8.5 | 3 | 0.04 |
|  |  |  |  |  |  |
| rel | spray | replicate | 4.3 | 2 | 0.11 |
|  |  | sex | 0.5 | 1 | 0.48 |
|  |  | replicate x sex | 0.4 | 2 | 0.81 |
|  |  |  |  |  |  |
| c564 x rel RNAi | spray | replicate | 22.5 | 2 | **<0.0001** |
|  |  | sex | 2.5 | 1 | 0.12 |
|  |  | replicate x sex | 0.1 | 2 | 0.96 |
|  |  |  |  |  |  |
| c564 x w1118 | spray | replicate | 8.4 | 2 | 0.02 |
| (driver control) |  | sex | 30.8 | 1 | **<0.0001** |
|  |  | replicate x sex | 4.0 | 2 | 0.14 |

*Drosophila* lines that contain mutated versions of Imd pathway genes were inoculated with *B. bassiana* (GHA strain) by spray and then monitored for survival for ten days post-inoculation (Figure 5). To confirm the lack of sexual dimorphism in relish mutants, relish RNAi construct flies were crossed to a fatbody driver (c564), inoculated with GHA and monitored for survival for 10 days post inoculation (Figure 6). To assess the contribution of replicate and sex to survival, we used a Cox Proportional Hazards model (Model D, see Materials and Methods) followed by an ANOVA. Where sex x replicate is significant, the strength of the phenotype varies among the replicates, as opposed to the dimorphism disappearing in some replicates (data not shown).
